# Lesion in Scalp and Skull as the First Manifestation of Hepatocellular Carcinoma

**DOI:** 10.1155/2016/2897048

**Published:** 2016-06-14

**Authors:** V. R. Ferraz, J. L. Vitorino-Araújo, L. Sementilli, J. F. Neto, J. C. E. Veiga

**Affiliations:** ^1^Neurosurgery Residency Program, Discipline of Neurosurgery, Santa Casa de Sao Paulo School of Medical Sciences, Dr. Cesario Motta Jr. 112, 01220-000 Sao Paulo, SP, Brazil; ^2^Discipline of Neurosurgery, Santa Casa de Sao Paulo School of Medical Sciences and Arnaldo Vieira de Carvalho Cancer Institute, Dr. Cesario Motta Jr. 112, 01220-000 Sao Paulo, SP, Brazil; ^3^Pathology Residency Program, Department of Pathology, Santa Casa de Sao Paulo School of Medical Sciences, Dr. Cesario Motta Jr. 112, 01220-000 Sao Paulo, SP, Brazil

## Abstract

Hepatocellular carcinoma (HCC) is the most common primary tumor of the liver and the fifth most common cancer in the world. The lungs, bone, and lymph nodes are frequent sites of metastasis of HCC. The purpose of the present study is show that metastases, although rare, must be among the differential diagnosis of skin lesions and that a diagnostic research based on these findings can be conducted. The authors report a rare case of metastatic hepatocellular injury to the scalp and skull treated by a radical surgical approach. Excision of the lesion in the scalp was performed “en bloc.” The tumor was supplied by the frontal branch of the superficial temporal artery. There are few case reports of metastatic HCC to scalp and skull; treatment of these lesions should be individualized in order to control symptoms, improve quality of life, and promote an increase in survival.

## 1. Introduction

Hepatocellular carcinoma (HCC) is the most common primary tumor of the liver and the fifth most common cancer in the world. It remains the fastest-growing cause of cancer death in men. The lung, bone, and lymph nodes are frequent sites of metastasis of HCC. The most common sites for distant skin metastasis of internal carcinomas are the chest, back, and abdomen [1].

In this paper we report a rare case of metastatic hepatocellular injury to the scalp and skull that underwent radical surgical approach with favorable outcome after six months.

## 2. Materials and Methods

In this paper we report a single rare case of metastatic hepatocellular injury to the scalp and skull treated by a radical surgical approach with good metastatic control. The patient and his family were informed about this publication and agreed to the publication of medical information about the patient. Immunohistochemistry was performed using antibodies against Hepatocyte (Dako®), polyclonal CEA (pCEA) (Neomarkers®), CD10 (Neomarkers), Villin (Neomarkers), CD34 (Neomarkers), TTF-1 (Zymed®), MOC-31 (Neomarkers), CK7 (Neomarkers), and CK20 (Neomarkers). The following tests were positive: Villin, pCEA, CD34, CK7, CD10, and Hepatocyte.

Dako is a manufacturer in Glostrup, Denmark, and Carpinteria, California, United States. Neomarkers Inc. is a manufacturer in Fremont, California, United States. Zymed Laboratories Inc. is a manufacturer in San Francisco, California, United States.

## 3. Results—Case Report

**Figure 1:**
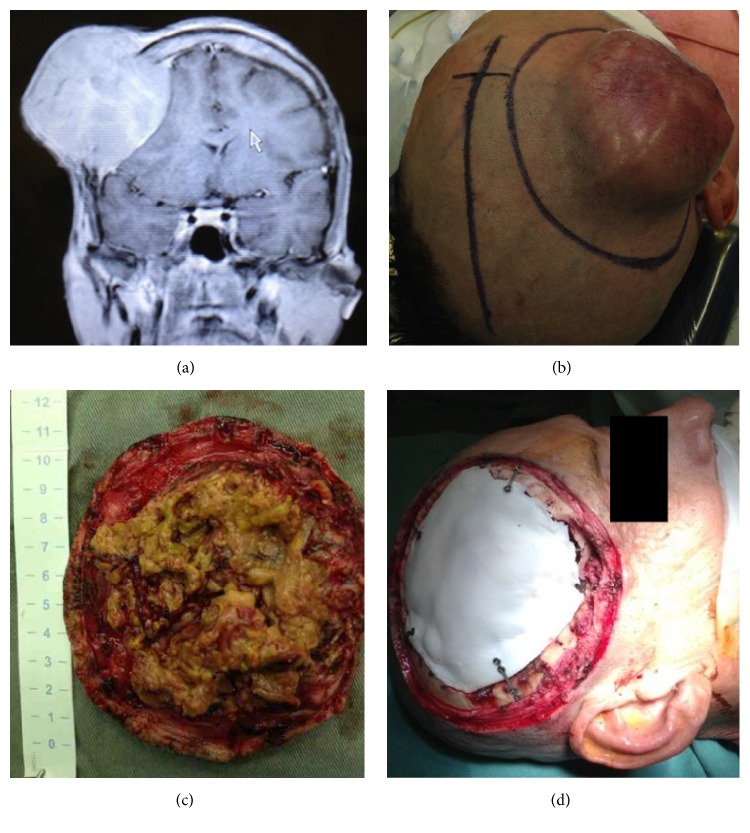
Intraoperative photos showing. (a) Brain MRI sequence with GD in T1 showing lesion with homogeneous enhancement contrast in scalp associated with bone invasion and compressive effect on the adjacent brain parenchyma. The mass enhanced homogeneously. (b) Delimitation of skin incision around the tumor on the right, midline delimitation on the left. (c) Tumor measures after the tumor resection. (d) Cranioplasty with bone cement.

The patient was a 53-year-old man who was previously healthy and who had no history of alcoholism or of Hepatitis C infection. The patient complained of having a bump on his head, with local pain. Preoperative magnetic resonance imaging (MRI) was performed and T1-weighted MRI sequences before and after administration of intravenous gadolinium demonstrated the mass in the scalp, subcutaneous tissues, and skull, compressing the underlying brain. The mass enhanced homogeneously (Figure 1(a)). Preoperative blood tests showed no abnormalities, and there was no coagulation disorders. He had a mass lesion in the right frontal region with 11 × 10 × 5 cm, adherence to deep tissue planes, and firm and rubbery consistency (Figures 1(b) and 1(c)). An “en bloc” resection of the scalp and skull mass, including the underlying dura to which it was adherent, via a larger frontal craniotomy, was performed. As suspected preoperatively the tumor was supplied by the frontal branch of the superficial temporal artery, confirmed during surgery; also increased local vascularization was observed.

**Figure 2:**
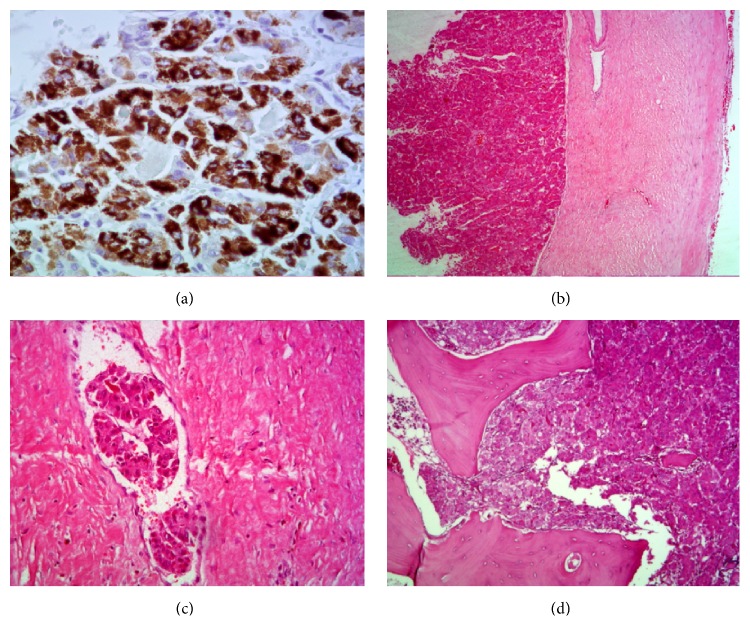
Photos of the hepatocarcinoma pathological structures and MRI image. (a) Hepatocyte. Typical hepatocellular carcinoma cells. 200x. (b) H/E. Tumor cells on the left and intact dura mater on the right. 25x. (c) H/E. Clumps of tumor cells within vascular spaces, demonstrating the tumor bloodstream dissemination. 100x. (d) H/E. Typical hepatocellular carcinoma. 100x.

We performed duraplasty with fascia lata and cranioplasty with appropriate bone cement (Figure 1(d)). The patient was discharged on the 10th postoperative day without motor deficits and in good condition. Histopathology showed free skin cancer margins, and the presence of clumps of cells within vascular spaces. There was no evidence of dural invasion; lesions were observed within small vessels and infiltrating bone structures (Figures 2(a), 2(b), 2(c), and 2(d)).

Histopathology showed neoplasm with characteristic of hepatocellular carcinoma, confirmed by immunohistochemistry. Villin, pCEA, CD34, CK7 and CD10, and Hepatocyte were positive as described above. Staining patterns were recorded for pCEA, CD10, and Villin as canalicular. CD34 staining of the endothelium surrounding the tumor cells in HCC was considered as positive and indicative of hepatocytic differentiation and in this case demonstrated sinusoidal CD34 staining. CD10 staining, like pCEA, is canalicular in HCC and normal liver, whereas cytoplasmic, apical, or membranous expression is observed in other tumors; canalicular CD10 expression was noted in this case. Hepatocyte is expressed in normal liver tissue and is formed against an unknown cytoplasmic or mitochondrial epitope in hepatocytes; in our case Hepatocyte was positive.

After the definitive diagnosis of metastatic lesion the patient was properly investigated for the screening of the primary tumor and then diagnosed with hepatocellular carcinoma after liver biopsy. Pathologically the lesion presented single, well-demarcated, nonencapsulated tumor with a fibrous band infiltrating throughout. On microscopic examination, the tumor was composed of well-differentiated polygonal cells that grow in nests and were separated by parallel lamellae of dense collagen bundles. The patient received adjuvant radiotherapy for brain postoperatively in order to control the metastatic lesion. Bone scintigraphy was performed and extracranial metastases were found in right clavicle, sternum, hip, and lumbar spine. After 6 months of the diagnosis the patient died from liver failure.

## 4. Discussion

Skull metastasis of HCC is relatively rare, in contrast to the incidence of skull metastasis in lung, breast, thyroid, and prostate cancers [2, 3]. In the past, the low survival rates for patients with HCC have resulted in low incidences of symptomatic extrahepatic metastases, such as lung and bone metastases (0–5%) [4, 5]. More recent studies have reported that the incidence of bone metastasis from HCC increased to 13% and that the most commonly involved sites were the vertebra, pelvis, rib, and skull [6]. Most HCC patients die of liver failure or internal bleeding without developing clinically apparent extrahepatic metastases [7].

Subcutaneous metastases are extremely rare and the majority of subcutaneous metastases appear to originate from needle tracks or surgical wound contamination [8]. Although subcutaneous metastasis is unusual, it can be the initial presenting sign of HCC [9]. Cutaneous metastases of HCC may appear as rapidly growing nodules on the scalp, chest, or shoulder. They may be single or multiple, firm, painless, nonulcerative, and reddish blue nodules, typically 1 to 2.5 cm. They may present similarly to basal cell carcinoma [10].

The hemorrhagic nature of metastatic HCC has been widely reported; patients with HCC and cirrhosis have declining liver function that affects the regulation of hemostasis on many levels. The decreased production of coagulation factors and anticoagulation proteins disturbs the balance of hemostasis and can lead to both hypercoagulable and coagulopathic states. Thrombocytopenia due to increased splenic sequestration from splenomegaly is too described [10–12]. This feature can lead to excessive bleeding during surgical procedures; the neurosurgeon should therefore be aware of this possible intraoperative complication.

The survival of patients with HCC and subcutaneous metastasis is not statistically different from those patients with HCC referred to radiation therapy for other reasons and there is no evidence that subcutaneous metastasis was associated with a poor prognosis in patients with HCC; thus an aggressive treatment of these lesions is justified [9, 10].

The HCC metastasis in the scalp with bone invasion are hypervascular and exceptionally rare. Probably spreading occurs through the blood to the final destination branches of the external carotid artery with consequent local deployment [13].

## 5. Conclusion

There are few case reports about skull and scalp hepatocellular carcinoma metastasis that emphasize the surgical management; however it is accepted that the surgical procedure is the most appropriate initial treatment option followed by adjuvant radiotherapy. Close attention should be paid to the coagulation status of the patient, as blood dyscrasia leading to excessive blood loss during surgical procedures can occur. Treatment of these lesions should be individualized in order to control symptoms, improve quality of life, and promote an increase in survival.
